# Effectiveness Assessment of a Multi-Functional Neonatal Incubator in the NICU

**DOI:** 10.3390/healthcare14070949

**Published:** 2026-04-04

**Authors:** Hyeonkyeong Choi, Wonseuk Jang

**Affiliations:** 1 Department of Medical Device Engineering and Management, Yonsei University College of Medicine, Seoul 06229, Republic of Korea; hyeonkyeong97@daum.net; 2 Medical Device Usability Research Center, Gangnam Severance Hospital, Yonsei University College of Medicine, Seoul 06230, Republic of Korea

**Keywords:** human factor, neonatal intensive care unit, multi-functional medical device, user experience, incubator, design process, usability

## Abstract

**Background/Objectives**: Preterm and critically ill neonates in neonatal intensive care units (NICUs) require multiple medical devices, including incubators, radiant warmers, phototherapy systems, and patient monitors. The coexistence of standalone devices without interoperability increases cognitive and operational burdens for healthcare providers and leads to spatial inefficiency. This study aimed to develop and evaluate a multi-functional neonatal incubator integrating these core functions into a single platform, using user-centered design (UCD) and usability engineering principles. **Methods**: By synthesizing and analyzing international standards (ISO 13485, IEC 62366-1, IEC 62366-2, and ISO 9241-210), a four-phase design process was established. Following the development of the monitoring system, the design was iteratively refined and validated through repeated formative usability evaluations. A summative usability evaluation was then conducted with 20 NICU clinicians in a simulated NICU environment, using 13 scenarios comprising 39 tasks. Outcome measures included task success rate, the After-Scenario Questionnaire (ASQ), the NASA Task Load Index (NASA-TLX), and the System Usability Scale (SUS). **Results**: The overall task success rate was 95.64%. When analyzed by function, success rates were 94.63% for incubator-related tasks, 98.33% for patient monitoring, 96.67% for radiant warmer tasks, and 98.33% for phototherapy tasks. The mean SUS score was 78.63, exceeding the benchmark score of 68 that indicates good usability. In addition, no statistically significant differences were observed in workload (NASA-TLX) or usability (SUS) scores according to clinical role or length of clinical experience. **Conclusions**: The multi-functional neonatal incubator developed in this study demonstrated high usability despite the integration of multiple medical device functions. The findings suggest that this integrated system has the potential to enhance clinical workflow efficiency, optimize spatial utilization, and improve patient safety in NICU settings.

## 1. Introduction

Neonatal incubators are medical devices designed to reduce the mortality rate of preterm infants and support their stable growth and development in an environment that mimics intrauterine conditions [1]. Over the past several decades, only minor improvements such as enhancements in lighting and cleaning features have been made to the user interface of incubators, while the overall system design and user experience have remained largely unchanged [1,2,3]. As of 2020, approximately 13.4 million babies, or about 10% of all births globally, were born prematurely. According to the World Health Organization (WHO), preterm birth refers to babies born before 37 weeks of gestation. Neonatal intensive care units (NICUs) provide critical care for preterm and medically fragile newborns [4,5,6]. From the moment of birth, neonates are assessed using multiple medical devices. When respiratory or thermoregulatory instability is present, radiant warmers are typically used in the delivery room. Once the preterm infant’s condition stabilizes, the infant is transferred to the NICU using a transport incubator. In the NICU, ongoing care commonly involves placement in an incubator or radiant warmer, administration of phototherapy for jaundice, and continuous physiological monitoring using patient monitoring systems.

Unlike other intensive care settings, NICUs are designed to accommodate admission, treatment, and surgical procedures for neonates, who represent a highly vulnerable patient population [7,8]. More than four independent devices are typically used concurrently, yet these devices function separately and lack interoperability. This fragmented device environment contributes to increased cognitive and operational burden for medical professionals. Moreover, spatial inefficiency resulting from the physical separation of devices leads to suboptimal use of limited NICU space. Consequently, clinical risks such as delayed emergency response and treatment are heightened.

In addition, preterm infants in NICU settings often present with complex clinical conditions that require continuous monitoring, coordinated interventions, and intensive nursing care [9]. Previous pediatric studies have highlighted that children with higher clinical complexity tend to require more frequent assessments, multidisciplinary coordination, and prolonged hospitalization [9]. This increased level of care complexity further amplifies the cognitive and operational burden on healthcare professionals, particularly in environments where multiple independent medical devices must be managed simultaneously [9]. Despite the widespread use of multiple devices in NICUs, there remains a lack of system-level integration and user-centered design approaches that address the combined clinical, operational, and environmental challenges in real-world settings.

To address these issues, user-centered design (UCD) principles have recently been adopted in the development of medical devices to reduce cognitive workload and optimize workflow through integrated device interfaces [10]. UCD emphasizes the incorporation of user needs, goals, and tasks throughout the design process, focusing on the end users [10,11,12,13]. As defined in ISO 9241-210, UCD is an iterative and interactive process aimed at improving usability and safety by considering user requirements and context of use during the design and evaluation phases [10,12,13,14]. Furthermore, design approaches that incorporate usability considerations can significantly reduce the likelihood of use errors and adverse events associated with medical devices [15]. The importance of usability has been globally recognized as a mandatory regulatory requirement in medical device approval and certification processes, and usability activities are systematically conducted in accordance with the usability engineering process defined in IEC 62366-1 [16,17]. Based on ISO 9241-210 and IEC 62366, iterative evaluation and design refinement contribute to improvements in the usability and operational efficiency of medical devices [18]. In high-risk environments such as NICUs, where multidisciplinary medical teams care for vulnerable neonates, a usability engineering approach based on human factors engineering (HFE) and UCD is essential. A review of prior incubator development efforts based on user-centered principles revealed a lack of integration with patient monitoring systems [1]. Most previous designs focused on creating incubators that are convenient for various user groups, including healthcare providers, neonates, and families [1]. Some examples include the use of circular beds and domed lids to resemble the uterus, or implementing rotating bed structures [1]. However, these designs failed to progress to commercialization [1]. According to previous studies, conventional incubators primarily focused on improving single functions such as temperature and humidity control [19]. In contrast, our approach aims not only to enhance individual performance but also to address the complexity of the clinical environment by integrating multiple functions into a single device. These limitations of previous studies have been translated into structural challenges in real NICU settings, where multiple independent medical devices must be used concurrently.

As a result, the simultaneous use of several standalone devices increases the workload of nurses and contributes to significant spatial inefficiency in the already limited NICU environment. Consequently, there is a growing need to develop new medical devices that can streamline admission and treatment processes for preterm infants and enhance clinical workflow efficiency. The multi-functional neonatal incubator is designed as an integrated system that combines the functionalities of a transport incubator, an incubator, a radiant warmer, a patient monitoring system, and a phototherapy unit into a single device. In this configuration, the conventional incubator display and the monitor used for measuring the infant’s vital signs are consolidated into a unified user interface to streamline clinical workflow and enhance usability.

**Figure 1:**
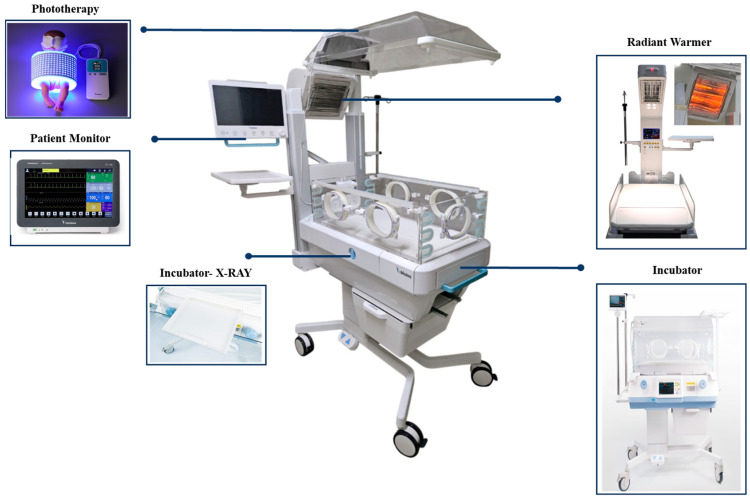
Multi-functional neonatal incubator.

This study contributes to the field by proposing a system-level, user-centered design approach for integrating multiple medical devices in NICU environments and by empirically validating the usability and effectiveness of the proposed system. Accordingly, this study aims to develop a multi-functional neonatal incubator (Figure 1) that integrates a warming unit, phototherapy function, and patient monitoring system into a single device primarily intended for preterm infants in the NICU. Throughout the development process, UCD principles and the HFE framework were applied to ensure usability and practical applicability within real NICU environments. Furthermore, a summative evaluation involving the intended users was conducted to assess the clinical feasibility and design validity of the proposed multi-functional medical device.

## 2. Materials and Methods

**Figure 2:**
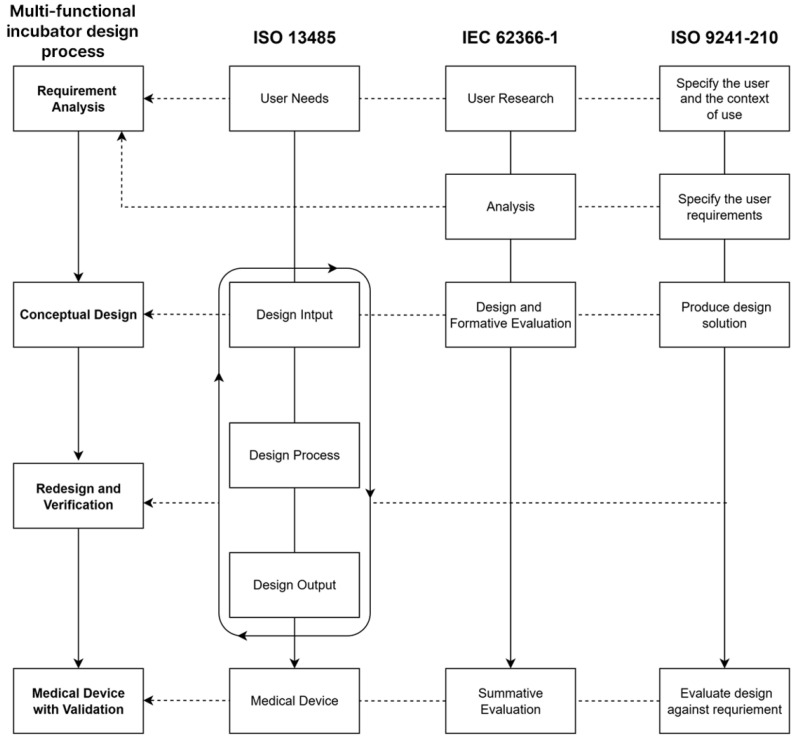
A user-centerd design framework for multi-functional incubator development based on international standards.

The identification and application of relevant standards and guidelines is essential in the design and development of medical devices. The selection of these standards was guided by their relevance to the medical device development lifecycle, their applicability to usability engineering and human-centered design principles, and their recognition within major international regulatory frameworks. In this study, the key processes of four international standards related to design were comparatively analyzed and synthesized to establish a unified four-phase design process for the development of a multi-functional neonatal incubator, as illustrated in Figure 2.

ISO 13485:2016, a quality management standard for medical devices, outlines a five-phase development process consisting of user needs, design input, design process, design output, and the final medical device itself [20,21]. IEC 62366-1, which focuses on usability engineering, is based on human-centered design principles and defines a four-step process: user research, analysis, design and formative evaluation, and summative evaluation [22,23]. In addition, ISO 9241-210 defines the human-centered design (HCD) process as comprising four iterative stages: specifying the user and context of use, specifying user requirements, producing design solutions, and evaluating the design against requirements [10]. Importantly, this process is not linear but emphasizes an iterative approach to design [10].

To design the multi-functional neonatal incubator, this study structured the overall design process into four phases: (1) requirement analysis, (2) conceptual design, (3) redesign and verification, and (4) medical device with validation. In particular, the first phase, “requirement analysis,” integrates key elements emphasized across four international standards, including elicitation of user needs, user research, contextual analysis, and specification of design requirements. The second phase, “conceptual design,” involves the visualization and structuring of initial design ideas with a focus on user-centered principles. The third phase, termed “redesign and verification,” encompasses iterative evaluations and design refinements aimed at improving usability and performance. The final phase, “medical device with validation,” refers to the development of a prototype and the execution of summative usability evaluation to verify the validity and usability of the final medical device. Based on these standards, this study developed a design process for the multi-functional neonatal incubator, with each phase structured in accordance with usability engineering principles. The composition of the four-phase design process for the development of the multi-functional incubator is presented in Table 1.

### 2.1. Requirements Analysis

To develop the multi-functional neonatal incubator, a user requirements analysis was conducted with medical staff working in NICUs. The survey involved eight healthcare professionals (six physicians and two nurses) with at least one year of NICU experience. A total of 22 user requirement items were derived through literature review and brainstorming sessions and were evaluated in terms of their perceived importance and frequency of use. The evaluation was conducted using a five-point Likert scale questionnaire (1 = not important at all/never used, 5 = very important/frequently used). The responses were analyzed using descriptive statistics (mean and standard deviation) to identify and prioritize the most critical user needs.

### 2.2. Conceptual Design

**Figure 3:**
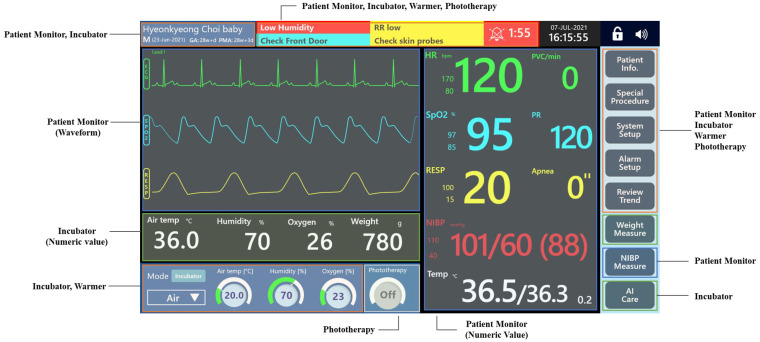
Multi-functional neonatal incubator graphical user interface.

To develop external concepts that reflected user requirements, initial idea sketches were created. A total of 12 design sketches were generated and subsequently evaluated through a survey involving 35 participants, including NICU medical staff and relevant stakeholders. The proposed external concepts were designed to integrate multiple functions such as patient monitoring, incubator, and radiant warmer into a single device in accordance with the identified user requirements. Each participant was asked to select their first and second preferred designs to determine overall preferences. To design the multi-functional neonatal incubator, approximately 350 user interface (UI) screens were developed using the prototyping tool Adobe XD. The screen design incorporated essential functions of conventional patient monitoring systems such as patient registration, alarm message display, waveform visualization, and parameter monitoring. These were integrated with the core incubator functions, including mode switching, weight measurement, oxygen and humidity control, and phototherapy. The initial design approach involved organizing the interface into functional zones. These zones were subsequently refined based on input from the primary users, namely medical professionals working in NICUs. The initial framework was structured to reflect the typical layout of patient monitors commonly used in NICU settings, in order to enhance familiarity and usability in clinical practice. Based on this foundation, specific interface components were incrementally developed. The final version of the main screen is presented in Figure 3, with detailed descriptions provided in Table 2.

Figure 3 presents the GUI integrated into the monitor of the multi-functional neonatal incubator shown in Figure 1. This interface consolidates the screen used for controlling conventional incubator parameters (e.g., humidity, temperature, and weight) with the display used for monitoring the infant’s vital signs. To support efficient user interaction, each vital sign waveform is presented together with its corresponding parameter value, and key incubator parameters such as humidity, oxygen concentration, and weight are displayed within the same integrated interface. The interface also enables mode transitions between the incubator and the radiant warmer through the lower-left functional area, allowing users to adjust thermal management settings with ease. A menu bar positioned on the right side provides direct access to essential functions to enhance navigability. Given that each incubator is assigned to a single infant, the upper-left section of the screen was designed to prominently display the infant’s identifying information.

### 2.3. Redesign and Verification

A total of 15 formative evaluations were conducted throughout the development process of the multi-functional medical device. The graphical user interface (GUI) of the multi-functional incubator underwent eight formative usability evaluations, through which iterative design modifications were made. A range of usability evaluation methods was employed, including expert reviews, focus group interviews, surveys, and advisory panel consultations. Based on the results of these evaluations, the detailed menu structure and interface components were continuously refined and updated. The evaluators consisted of neonatologists and nurses from the Neonatal Intensive Care Unit (NICU) at Severance Hospital, as well as usability engineering experts. Their feedback and requirements were systematically incorporated into each stage of the design iteration.

### 2.4. Medical Device with Validation

#### 2.4.1. Participants

A total of 20 medical professionals working in NICUs participated in the summative evaluation. The participants included five physicians and fifteen nurses, all of whom had at least one year of clinical experience in the NICU. The average age of all participants was 37.4 years, with physicians averaging 44.8 years and nurses 34.93 years. Nineteen participants were female and one was male. The average professional experience in the relevant clinical field was 8.55 years, ranging from 2 to 18 years.

The study was approved by the Institutional Review Board (IRB) of Gangnam Severance Hospital, Yonsei University Health System (IRB No. 3-2022-0145), and was conducted in accordance with institutional guidelines and ethical standards. Informed consent was obtained from all participants prior to their involvement in the study.

#### 2.4.2. Study Procedures

**Figure 4:**
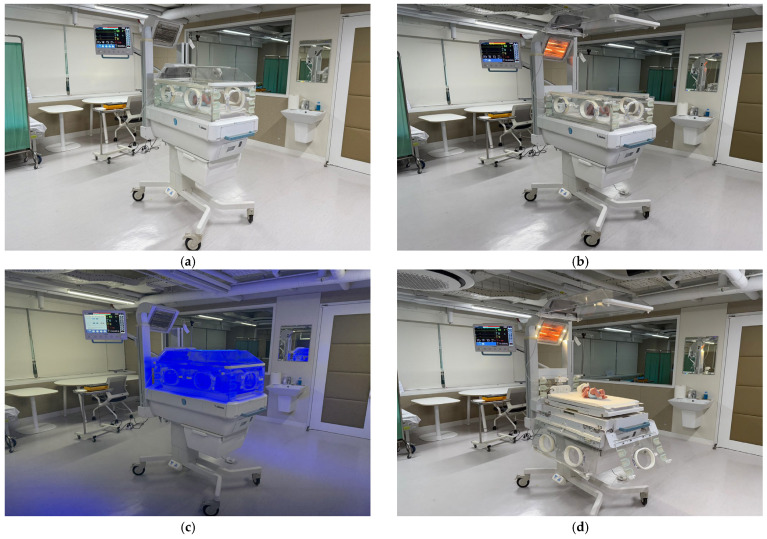
Evaluation environment of the multi-functional neonatal incubator under different operational modes: (**a**) incubator mode; (**b**) radiant warmer mode; (**c**) phototherapy mode; and (**d**) surgical mode.

The summative evaluation was conducted in a simulated environment designed to reflect the intended use environment of the multi-functional neonatal incubator, specifically resembling the conditions of a NICU. As shown in Figure 4, the evaluation environment was configured to replicate clinical conditions in a NICU. To represent the intended use scenarios, four operational modes of the multi-functional neonatal incubator, including incubator mode, radiant warmer mode, phototherapy mode, and surgical mode, were systematically incorporated into the evaluation setup.

**Figure 5:**
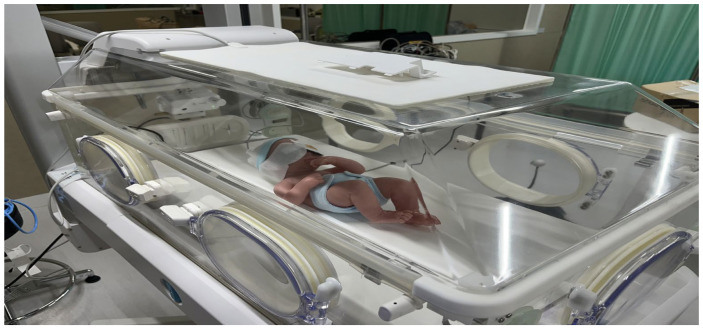
Neonatal simulation mannequin used for evaluation inside the incubator.

As presented in Figure 5, a neonatal simulation mannequin was employed for the evaluation to ensure consistency and safety while accurately representing clinical use conditions. The evaluation scenarios were structured to reflect actual NICU workflows, including placement of the simulated patient in the incubator and administration of phototherapy as part of routine clinical care. During phototherapy scenarios, an eye shield was applied to the mannequin in accordance with standard clinical practice to protect the infant’s eyes, thereby closely approximating real patient conditions.

To replicate the clinical setting, the evaluation room was maintained at a temperature range of 22 °C to 26 °C and a relative humidity of 30% to 60% prior to the start of the evaluation. The moderator provided participants with a scenario-based evaluation, including a pre- evaluation briefing on the purpose of the evaluation and instructions on how to use the device.

A separate observer (data analyst) monitored and recorded the entire evaluation process remotely from an observation room using Blackmagic Media Express software (version 1.0). Following the usability testing, participants completed three standardized questionnaires: the After-Scenario Questionnaire (ASQ), NASA Task Load Index (NASA-TLX), and the System Usability Scale (SUS), as summarized in Table 3.

The ASQ, developed by Lewis and based on the ISO 9241-11 usability standard, assesses user satisfaction regarding ease of use, efficiency, and adequacy of information using a seven-point Likert scale [24,25,26]. The NASA-TLX measures subjective workload across six subscales: mental demand, temporal demand, physical demand, performance, effort, and frustration. Each item is rated on a 21-point scale [27,28,29]. The SUS is one of the most widely used instruments for evaluating overall system usability and consists of 10 items rated on a five-point Likert scale [30,31,32,33].

#### 2.4.3. Data Analysis

A summative usability test was conducted on the final prototype of the multi-functional neonatal incubator developed in this study. Each task, based on predefined use scenarios, was classified into one of three outcome categories: Completed, Completed with issues, or Non-completed. Task success rates were then calculated based on these classifications.

Objective usability measures were analyzed using SPSS version 27 (IBM Corp., Armonk, NY, USA), focusing on the results of ASQ, NASA-TLX, and SUS. For the ASQ, mean and standard deviation values were used to evaluate user satisfaction regarding ease of use, efficiency, and the adequacy of provided information.

NASA-TLX scores were converted to a 0–100 scale by subtracting 1 from each raw score and multiplying by 5, where lower scores indicate a lower perceived workload [34]. The overall workload scores were interpreted as follows: low (0–9), moderate (10–29), somewhat high (30–49), high (50–79), and very high (80–100). Notably, for the ‘Performance’ subscale in the NASA-TLX, a lower score reflects a higher perceived performance in task execution.

SUS scores were calculated by subtracting 1 from the responses to odd-numbered items and subtracting the responses from 5 for even-numbered items. The adjusted scores were then multiplied by 2.5 and summed to yield a total score ranging from 0 to 100. To compare differences in workload and overall system usability according to participants’ clinical role and clinical experience, a non-parametric Mann–Whitney U test was performed. Descriptive statistics were presented as means and standard deviations, and the significance level was set at *p* < 0.05. The significance level was set at *p* < 0.05. In addition to *p*-values, effect sizes (r) were calculated for Mann–Whitney U tests using the formula r = Z/√N, where N represents the total number of observations [35]. The magnitude of effect sizes was interpreted based on commonly used benchmarks, where values of r < 0.10 were considered trivial, 0.10–0.29 small, 0.30–0.49 medium, and ≥ 0.50 large [35].

## 3. Results

### 3.1. Results of User Requirements Analysis

An analysis of the 22 identified user requirements revealed that the overall mean importance score was 3.6, while the mean frequency of use score was 3.2. Among the key requirements, participants expressed a strong need for the ability to view both patient monitor parameters and incubator-related data on a single integrated display. Additionally, there was a demand for a function that allows the monitoring of weight trends measured by the incubator directly from the monitor.

The two items with the highest importance scores were the Apnea Intervention Alarm and the drawer for the X-ray detector located beneath the incubator, each with an average importance rating of 4.80 (SD = 0.45). The corresponding frequency of use scores were 4.80 (SD = 0.45) and 4.40 (SD = 0.89), respectively.

In particular, for the Apnea Intervention Alarm, participants emphasized the need for direct recognition and control of apnea alarms within the incubator system. In the conventional setup, clinical staff must first respond to alarms detected through the patient monitoring system and then physically move to the incubator to act. This feedback indicates a clear need for an embedded interface that enables immediate alarm recognition and intervention from within the incubator itself.

### 3.2. Design and Verification Process

**Figure 6:**
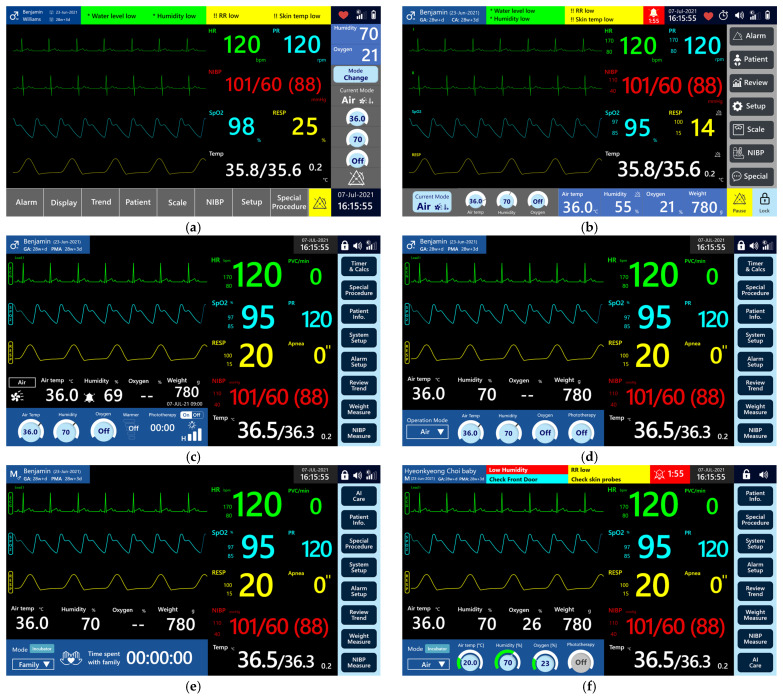
Design iteration process of multi-functional neonatal incubator: (**a**) conceptual design; (**b**) 1st iteration; (**c**) 2nd iteration; (**d**) 3rd iteration; (**e**) 4th iteration; (**f**) final design.

The graphic user interface design process, which was refined through eight formative evaluations. The representative screen layouts reflecting each design iteration are shown in Figure 6, following the summary of evaluation methods and user feedback presented in Table 4. Overall, the formative evaluations demonstrated a strong preference for maintaining interface consistency with conventional patient monitoring systems, simplified menu architectures, and the prioritized placement of clinically frequent functions. Across successive iterations, user feedback consistently underscored the importance of readability at a distance, minimization of visual clutter, and rapid access to safety-critical controls in NICU environments. These findings systematically guided the progressive refinement of the GUI design.

In the initial conceptual design (Figure 6a), the patient monitoring menu was positioned at the bottom of the screen, while the incubator-related menu was arranged vertically on the right side, similar to conventional patient monitoring systems. The central area displayed waveforms and numerical values of physiological parameters. However, formative evaluation indicated a preference for locating the primary menu on the right side. Accordingly, in the 1st Iteration design (Figure 6b), incubator mode buttons were moved to the bottom, and parameter adjustment functions were relocated to the right menu.

To enhance menu visibility and usability of the bottom buttons, the 2nd Iteration design (Figure 6c) simplified button layouts. Evaluation feedback suggested that detailed level adjustments for the radiant warmer and phototherapy modes should be provided in sub-screens rather than on the main screen, where only On/Off control should be retained to optimize usability. This was reflected in the 3rd Iteration design (Figure 6d), in which the bottom menu tabs were streamlined.

In the 4th Iteration design (Figure 6e), an evaluation was conducted to determine the optimal ordering of menu buttons based on user preferences. The results informed a reorganization of the menu structure, in which functions with higher clinical frequency were placed in priority order and the measurement button was relocated to the bottom of the screen. Additionally, a dedicated screen to support kangaroo care in the NICU was introduced, and based on clinicians’ feedback, the mode name was revised to “Family mode.” The final design (Figure 6f) further enhanced user perception by incorporating color-coded visualization of incubator setting adjustments.

### 3.3. Summative Evaluation of Multi-Functional Neonatal Incubator

#### 3.3.1. Participants Analysis

A total of 20 medical professionals participated in the summative usability evaluation. Table 5 presents the demographic characteristics of participants who took part in the summative evaluation. All participants had at least one year of clinical experience in the neonatal intensive care unit (NICU) at Severance Hospital. The average age of the participants was 37.4 years, and the average duration of professional experience was 8.55 years. The group consisted of 19 females and 1 male. The participants consisted of five physicians and fifteen nurses.

All participants had a minimum of one year of hands-on experience with relevant NICU equipment, including patient monitors, incubators, radiant warmers, and phototherapy devices. All participants had experience using the individual devices in the NICU; however, none had prior experience operating them as a single integrated system.

#### 3.3.2. Usability Test and Survey Analysis

**Figure 7:**
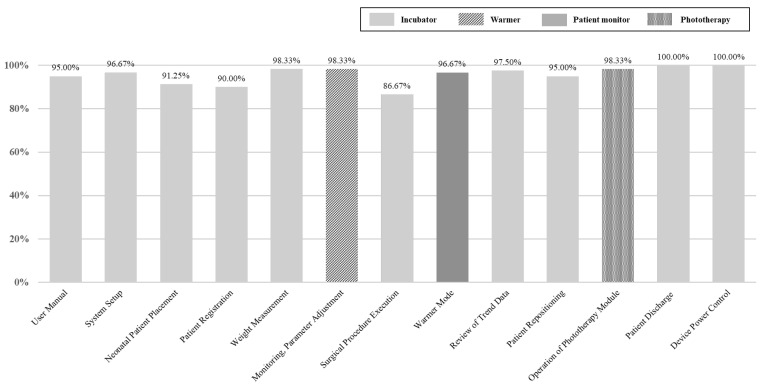
Scenario-Based Success Rates Across Integrated Device Functions.

The usability evaluation consisted of 13 scenarios and 39 detailed tasks, yielding an overall mean success rate of 95.64% (Table 6 and Figure 7).

When analyzed by function, the success rates were 94.63% for incubator-related tasks, 98.33% for patient monitoring, 96.67% for the radiant warmer, and 98.33% for phototherapy (Table 7). These findings indicate that, despite the multifunctional integration of the multi-functional medical device, consistently high success rates were achieved across all functional domains.

Table 7 presents a comparison of task success rates by device functions according to clinical role (physicians and nurses) and NICU experience. Overall task success was slightly higher among nurses (95.73%) than physicians (94.00%). When stratified by clinical experience, participants with ≥8 years of NICU experience demonstrated a higher overall success rate (96.15%) compared with those with <8 years (94.62%), while high performance was consistently maintained across both experience groups.

At the functional level, nurses achieved higher success rates than physicians for the incubator, patient monitoring, and radiant warmer functions, whereas physicians demonstrated higher success rates for phototherapy tasks. With respect to clinical experience, participants with ≥8 years of experience showed higher success rates for the incubator and patient monitoring functions. For the radiant warmer function, both experience groups exhibited identical success rates (96.67%). In contrast, for the phototherapy function, participants with <8 years of NICU experience achieved a 100% task success rate.

As presented in Figure 7 and Table 6 and Table 7, the representative scenarios for each function are as follows: scenario 6 (Monitoring and Parameter Adjustment) for patient monitoring, scenario 8 (Warmer Mode) for the radiant warmer, and scenario 11 (Operation of Phototherapy Module) for phototherapy. All other scenarios were classified as incubator-related functions. Among the 13 scenarios, scenario 7 (Surgical Procedure Execution) demonstrated the lowest success rate. In particular, the task of turning on the surgical light showed the highest failure rate. Although this function was accessible via the “Operation” tab under the “System Setup” menu, many participants attempted to access it through the “Special Procedure” menu and were unable to complete the task. This outcome likely reflects clinical practice in NICUs, where external shadowless lamps are typically used for surgical procedures, making the integrated surgical light unfamiliar and its access pathway less intuitive. Nevertheless, subjective satisfaction with the function was high, with mean scores of 6.70 ± 0.46 for ease of use, 6.75 ± 0.43 for efficiency, and 6.75 ± 0.43 for adequacy of information. Detailed results are presented in Table 6.

**Figure 8:**
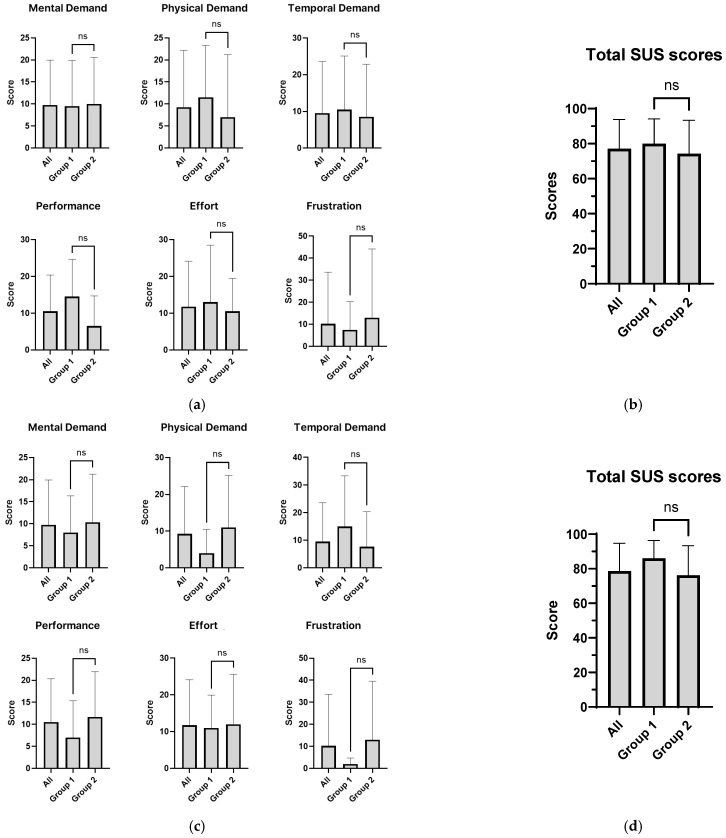
Comparison of task workload (NASA-TLX) and system usability (SUS) according to clinical role and NICU experience; (**a**) task workload scores by group 1 (physicians) and group 2 (nurses); (**b**) sus scores by group 1 (physicians) and group 2 (nurses); (**c**) task workload scores by group 1 (≥8 years) and group 2 (<8 years); (**d**) sus scores by group 1 (≥8 years) and group 2 (<8 years). ns, not significant (*p* > 0.05).

The results of the analysis of task load and system usability satisfaction according to clinical role and NICU work experience are presented in Table 8 and Table 9 and Figure 8. First, participants were categorized by clinical role into a physician group (Group 1) and a nurse group (Group 2). To examine differences in task load and system usability between the two groups, non-parametric analyses using the Mann–Whitney U test were performed. As shown in Figure 8a, no statistically significant differences were observed across all six subscales of task load between physicians and nurses (all *p* > 0.05), indicating that perceived task load did not differ according to clinical role.

Next, participants were divided according to NICU experience into those with ≥8 years of experience (Group 1) and those with <8 years of experience (Group 2). The Shapiro–Wilk test indicated that the assumption of normality was not satisfied (*p* < 0.05); therefore, group comparisons were conducted using the Mann–Whitney U test. Similarly, no statistically significant differences were found across any of the six task load subscales between the two experience-based groups (all *p* > 0.05), suggesting that perceived task load was independent of years of NICU experience (Figure 8c).

The overall System Usability Scale (SUS) score was 78.63 (SD = 15.7), which exceeds the commonly accepted usability threshold of 68, indicating good overall usability. As shown in Figure 8b, no statistically significant difference in system usability scores was observed between physicians and nurses based on clinical role (*p* = 0.230). Similarly, as illustrated in Figure 8d, no significant difference was found between participants with ≥8 years and <8 years of NICU work experience (*p* = 0.739). Overall, system usability satisfaction was found to be independent of both clinical role and NICU work experience, demonstrating consistent usability across different user groups. Effect size estimates for the Mann–Whitney U tests were generally trivial to small. For comparisons by clinical role, effect sizes ranged from 0.040 to 0.274, indicating limited magnitude of between-group differences. For comparisons by NICU experience, most effect sizes were trivial, although physical demand (r=0.303) and performance (r=0.389) showed medium effect sizes despite the absence of statistically significant differences.

## 4. Discussion

### 4.1. Main Findings

Summative evaluation was conducted to assess the improved patient monitoring system. This study demonstrated that despite the integration of multiple functions into a single multi-functional neonatal incubator, usability remained high.

All integrated tasks demonstrated success rates exceeding 90%. In the early stage of user requirements analysis, the simultaneous presentation of patient monitoring parameters and incubator-related information on a single integrated display emerged as a critical requirement. Accordingly, a multi-functional device was developed to address this need, and usability evaluation results showing high task success rates verified that the design effectively met the identified user requirements. In particular, the Review of Trend Data task achieved a notably high success rate of 97.50%, underscoring the effectiveness of the system in supporting continuous patient monitoring in the NICU environment, where such functionality is critical. These findings suggest that functional convergence, when guided by a user-centered design approach, does not inherently compromise usability, even in complex NICU environments.

In the radiant warmer-related scenario (Warmer Mode), a task success rate of 96.67% was achieved. A task failure occurred during the transition from incubator mode to warmer mode, in which the participant raised the canopy without explicitly switching to warmer mode. This error can be attributed to prior experience with conventional incubators, where opening the canopy automatically activates the warmer. Based on this familiarity, the participant perceived the canopy-open function as equivalent to initiating warmer mode, resulting in a use error. This finding suggests that the observed error was driven by habituation to legacy devices rather than by interface deficiencies. Such errors are expected to be mitigated through increased exposure to the multi-functional device and appropriate user training. These findings also suggest that the system’s interaction logic for mode transition may not have been fully aligned with users’ established mental models based on conventional workflows, highlighting the importance of designing workflow-consistent interactions in integrated systems.

For surgical light–related tasks, task failures were primarily associated with differences in clinical workflow rather than interface design issues. In typical NICU practice, surgical procedures are often performed at the bedside using externally mounted shadowless lamps, making the integrated surgical light within the multi-functional incubator relatively unfamiliar to users. Consequently, some participants experienced difficulty locating or activating this function. These findings further suggest that the access pathway for the surgical light function may not have been fully aligned with users’ expectations shaped by conventional clinical workflows, highlighting the importance of intuitive navigation design in integrated systems. Despite these task failures, subjective usability ratings for this function were high, with mean ASQ scores of 6.70 (SD = 0.46) for ease of use, 6.75 (SD = 0.43) for efficiency, and 6.75 (SD = 0.43) for adequacy of information. These results indicate that, once recognized, the function was perceived as intuitive and satisfactory by users.

Analysis by clinical role indicated that, although physicians and nurses in the NICU routinely share and operate a wide range of medical devices, their primary workflows differ depending on clinical scenarios; both groups demonstrated consistently high task success rates. In general, physicians primarily engage in workflows related to patient monitoring and procedural or surgical interventions, whereas nurses are more frequently responsible for broader NICU operational workflows, including patient registration, device configuration, mode transitions, and system shutdown. Despite these differences in predominant workflows, the results of this study showed high success rates across all tasks, from patient registration to powering off the device, for both clinical roles. These findings suggest that the multi-functional incubator effectively supports diverse clinical workflows and can be used efficiently regardless of clinical role, highlighting its role-independent usability within the NICU environment. Similarly, task success rates were comparable across levels of NICU experience, suggesting that high performance was not limited to highly experienced users. Rather, these findings indicate that novice users were able to adapt to the system relatively quickly. Given that the individual functions integrated into the multi-functional incubator were already familiar to users in their standalone forms, functional convergence did not impose a substantial usability burden, allowing users to operate the integrated system without significant difficulty.

Furthermore, high levels of user satisfaction were observed across all three domains of the ASQ, including ease of use, efficiency, and adequacy of information. Analysis of the NASA-TLX revealed a low level of physical demand (9.25 ± 12.577), which can be attributed to the integrated design of the patient monitoring and incubator functions. This represents a meaningful improvement compared with conventional workflows, in which clinicians are required to access vital signs and weight information across multiple standalone devices. By enabling integrated access to key clinical information through a single display, users perceived a substantial enhancement in overall usability.

Importantly, the SUS item assessing whether “various functions were well integrated” also received a high mean score of 3.45 (SD = 0.92), validating the necessity of functional integration that was initially highlighted in the user requirements analysis. Clinically, these results are meaningful, as they suggest that the multi-functional incubator may enhance workflow efficiency and spatial utilization within NICUs by integrating traditionally separate devices into a single platform. Such integration could reduce the cognitive load of healthcare providers, enable faster recognition of critical alarms, and improve response times during emergencies. By simplifying device operation and minimizing the need to manage multiple independent systems, the multi-functional incubator has the potential to improve patient safety and overall quality of care for preterm and critically ill neonates.

The findings of this study suggest several potential implications for clinical practice in NICU settings. The integration of multiple functions into a single device may simplify clinical workflows by reducing the need to operate multiple standalone systems, particularly in time-sensitive situations where rapid access to patient information is critical. In addition, a unified user interface may support faster recognition of patient status and alarms, thereby potentially contributing to improved patient safety.

Furthermore, reducing the number of independent devices may help decrease the cognitive workload of healthcare professionals and improve operational efficiency, which is consistent with the low NASA-TLX scores observed in this study. From a cost and resource utilization perspective, integrating multiple devices into a single platform may enable more efficient use of NICU space and reduce equipment redundancy and maintenance requirements.

### 4.2. Strengths and Limitations

First, this study involved the active participation of researchers from the early stages of the multi-functional incubator’s development, enabling the incorporation of user requirements into the design. This approach facilitated a user-centered design process and ensured that both the development and evaluation phases reflected actual clinical environments.

Second, the usability evaluation included both physicians and nurses, the primary user groups who operate equipment in NICUs. By involving multiple professional groups, the study was able to capture diverse perspectives on device use. However, the evaluation was conducted with only 20 medical professionals from a single institution (Severance Hospital), which may limit the generalizability of the findings. Nevertheless, in usability engineering, sample size is determined based on the ability to identify use-related problems rather than to achieve statistical generalization. According to IEC TR 62366-2 (Annex K), relatively small sample sizes are sufficient to detect the majority of usability issues, and the sample size used in this study is consistent with these recommendations. Despite this, the single-center setting may still limit external validity, and future multicenter studies involving more diverse user populations are warranted.

Third, this study is significant, as it represents one of the first usability evaluations of a multi-functional neonatal incubator, a device that is rarely available in commercial practice. In particular, the integration of multiple standalone devices, including patient monitors, incubators, radiant warmers, and phototherapy systems, into a single system was evaluated directly from the user’s perspective, which constitutes a major strength of this research.

Fourth, the study proposed and applied a dedicated design process tailored to the development of multi-functional devices. This framework provides a systematic methodology that can be adapted for the development of other multi-functional medical devices in the future.

Fifth, instead of relying on a single metric, the usability evaluation employed multiple complementary measures, including task success rate, the ASQ, NASA-TLX, and SUS. This multidimensional approach strengthened the reliability of the usability findings.

Nevertheless, several limitations should be noted. The usability evaluation was conducted in a simulated environment rather than in an actual NICU setting and thus may not fully reflect real-world clinical applicability. These limitations were partially mitigated by conducting the usability evaluation in accordance with established usability engineering standards (IEC 62366-1), which recommend simulation-based testing to safely identify use-related problems prior to clinical application. In addition, participants were recruited from intended user groups with relevant clinical experience, ensuring that the evaluation reflected realistic clinical workflows. Clinical trials involving real patients are needed to validate the device under actual operating conditions. Furthermore, additional research will be required after commercialization to examine the device’s long-term usability and performance in routine clinical environments. To further address safety considerations, a simplified use-related risk analysis based on the usability findings has been provided in Appendix A.

## 5. Conclusions

This study demonstrated the feasibility and usability of a multi-functional neonatal incubator that integrates an incubator, radiant warmer, phototherapy unit, and patient monitoring system into a single platform. By applying a structured user-centered design process and iterative formative evaluations, the device was optimized to meet clinical user needs and achieved high usability in summative testing conducted in a simulated environment.

Clinically, the multi-functional incubator has the potential to streamline workflows, improve spatial efficiency, and reduce the cognitive burden on NICU staff. In addition, usability evaluation can support the identification of workflow-related challenges and potential use-related errors that may arise in complex NICU environments. Together, these aspects suggest that the system may contribute to more effective care for preterm and critically ill neonates. Methodologically, the study highlights the value of a dedicated multi-functional design process that may be adapted for the development of other multifunctional medical devices.

## Figures and Tables

**Table 1:** Overview of the design process for a multi-functional neonatal incubator.

Steps		Activities	Outcomes
Requirements Analysis	Analysis of user requirements for the development of a neonatal incubator.	Conducted brainstorming sessions and user surveys involving 8 NICU clinicians.	Defined product concepts based on prioritization and frequency of identified user needs.
Conceptual Design	Design of the device appearance and graphical user interface based on user requirements.	Designed graphical user interfaces (GUI) using simulation tools and Adobe XD for UI/UX prototyping.	Developed mid-fidelity prototypes for GUI design.
Redesign and Verification	Usability evaluation and iterative redesign of the initial multi-functional medical device.	Formative evaluations were conducted based on feedback from end users and experts regarding the proposed design. Identified usability issues and iteratively refined the design.	Derived improved design proposals addressing usability issues.
Medical Device with Validation	Usability evaluation of the functional performance of the multi-functional medical device.	A total of 20 NICU clinicians participated in the overall scenario-based evaluation and survey of the multi-functional incubator.	A total of 20 NICU clinicians participated, providing usability feedback in a simulated clinical setting.

**Table 2:** Main screen components of the multi-functional neonatal incubator graphical user interface.

Category	Component	Details
Main layout	Patient monitor	The main screen was designed to follow the conventional monitor layout of existing patient monitoring systems, displaying both physiological waveforms and numerical parameter values.
Waveform	Patient monitor	Waveforms for commonly monitored neonatal vital signs, including ECG, SpO_2_, and RESP, are presented using the same waveform format as conventional patient monitoring systems.
Numeric value	Patient monitor, Incubator	The main screen displays numeric values from both the patient monitor (SpO_2_, PR) and the incubator (air temperature, humidity, oxygen concentration).
Patient Info	Patient monitor	These elements are typically positioned at the top of the screen, consistent with patient monitoring system conventions.
Menu button	Patient monitor, Incubator, Radiant warmer, Phototherapy	To accommodate the characteristics of all four integrated devices, menu buttons are positioned on the right side of the screen, while measurement buttons are located at the bottom.
Mode (bottom)	Incubator, Radiant warmer, Phototherapy	Based on incubator-specific operational characteristics, mode switching (incubator/warmer) and the on/off controls for the warmer and phototherapy are located in the lower-left area of the screen.
Alarm message	Patient monitor	In patient monitors, alarms are typically displayed at the center of the screen using color coding in accordance with alarm standards.
Status bar	Patient monitor	The lock icon and patient monitor sound icon are typically positioned in the upper-right corner.

**Table 3:** Evaluation metrics of the summative evaluation.

Evaluation Method	Description	Performance Metric	Result Analysis
Usability Test	A structured evaluation to assess how effectively and efficiently users perform predefined tasks with the system. Participants perform assigned tasks in a simulated NICU environment, guided by the test administrator, and task completion is assessed.	Task completion evaluation for 13 scenarios and 39 tasks categorized into three groups: completed (C), completed with issues (CI), not completed (NC)	The success rate was calculated for each scenario, with higher success rates indicating better usability.
ASQ	A post-task questionnaire developed by Lewis based on ISO 9241-11 to measure user satisfaction with system usability, focusing on ease of use, efficiency, and adequacy of provided information.	3 questions rated on a 7-point Likert scale (1 = strongly disagree, 7 = strongly agree) for each scenario.	The mean and standard deviation were calculated for each scenario.
NASA TLX	A subjective workload assessment tool developed by NASA to evaluate perceived workload during task performance across six dimensions: mental demand, physical demand, temporal demand, performance, effort, and frustration.	Each dimension is rated using a 21-point scale (0–100 after scaling).	Lower scores indicate lower perceived workload. Statistical analysis was performed using SPSS to compare differences based on participants’ years of experience.
SUS	A widely used 10-item questionnaire that provides a global view of system usability.	Items rated on a 5-point Likert scale (1 = strongly disagree, 5 = strongly agree); scored to yield a total from 0 to 100.	Scores above 68 are considered above average usability categorized as A (excellent) to F (poor). Statistical analysis was performed using SPSS to compare differences based on participants’ years of experience.

**Table 4:** Evaluation Methods and User Feedback Across the Design Iteration Process.

Process	Evaluation Method	User Feedback
Conceptual design	Expert review	The patient information display area was expanded to enhance visual clarity. The menu button was relocated to the upper-right corner to maintain consistency with existing medical devices and improve user familiarity. The clock was positioned at the top of the interface to support intuitive time recognition and overall usability.
1st Iteration	Advisory panel review	Each waveform was clearly labeled with its corresponding parameter to enhance visibility and interpretability. The number of icons was reduced to essential functions (e.g., lock, sound, and brightness) to minimize visual clutter. Because the menu bar combined icons and text, text legibility was reduced when viewed from a distance; therefore, the menu items were presented using text only to improve readability. Related icons were grouped and relocated to the upper-right corner to improve clarity and usability. The waveform parameters and corresponding numeric values were displayed on a single line to enhance clarity and readability.
2nd Iteration	Focus group interview	Mode changes (incubator and warmer) were presented using text labels rather than icons to enhance clarity and usability. For the warmer and phototherapy functions, on/off controls were provided on the main screen, while detailed settings were accessed through a separate menu.
3rd Iteration	Focus group interview	As the timer and calculation functions were infrequently used, the menu was reorganized based on actual clinical usage, with the measurement button placed at the bottom and frequently used functions positioned in the central and lower areas to reduce nurses’ workload. Because bar-only value displays may reduce visibility at a distance, color coding was incorporated to enhance clarity.
4th Iteration	Survey	The AI Care function is associated with monitoring growth indices and is closely related to weight measurement; therefore, positioning it in the lower area of the screen, in proximity to related functions, would be preferable.
Final design	Focus group interview	-

**Table 5:** Sociodemographic characteristics of the summative evaluation participants.

Variable	Option	Frequency
Clinical role	Physician	5
	Nurses	15
Age	20–29 years	5
	30–39 years	8
	40–49 years	6
	50–59 years	1
Department of participants	Neonatal Intensive Care Unit(NICU)	20
Manufacturer name (incubator)	GE	13 ^1^
	Drager	18 ^1^
Use experience with similar devices (incubator)	Less than 3 years	3
	More than 3 years, less than 5 years	2
	More than 5 years, less than 10 years	9
	More than 10 years	6
Manufacturer name (patient monitor)	GE	13 ^1^
	Phillips	12 ^1^
	Drager	13 ^1^
Use experience with similar devices (patient monitor)	Less than 3 years	2
	More than 3 years, less than 5 years	2
	More than 5 years, less than 10 years	9
	More than 10 years	7

^1^ Repetition is acceptable.

**Table 6:** Summative evaluation results for each scenario.

No	Use Scenario	Success Rate	ASQ1	ASQ2	ASQ3
1	User Manual	95.00%	5.60 ± 1.16	5.40 ± 1.36	5.95 ± 0.97
2	System Setup	96.67%	6.80 ± 0.54	6.38 ± 1.10	6.65 ± 0.61
3	Neonatal Patient Placement	91.25%	6.70 ± 0.46	6.75 ± 0.43	6.75 ± 0.43
4	Patient Registration	90.00%	6.70 ± 0.56	6.60 ± 0.58	6.65 ± 0.57
5	Weight Measurement	98.33%	6.30 ± 0.95	6.05 ± 1.43	6.20 ± 1.08
6	Monitoring, Parameter Adjustment	98.33%	6.65 ± 0.57	6.65 ± 0.48	6.60 ± 0.49
7	Surgical Procedure Execution	86.67%	6.70 ± 0.46	6.75 ± 0.43	6.75 ± 0.43
8	Warmer Mode	96.67%	6.20 ± 1.03	6.25 ± 1.09	6.25 ± 0.89
9	Review of Trend Data	97.50%	6.60 ± 0.58	6.55 ± 0.67	6.50 ± 0.67
10	Patient Repositioning	95.00%	5.80 ± 1.36	6.00 ± 1.26	5.80 ± 1.40
11	Operation of Phototherapy Module	98.33%	6.50 ± 0.92	6.50 ± 1.12	6.45 ± 1.12
12	Patient Discharge	100.00%	7.00 ± 0.00	7.00 ± 0.00	7.00 ± 0.00
13	Device Power Control	100.00%	7.00 ± 0.00	7.00 ± 0.00	7.00 ± 0.00
	Overall	95.64%	6.50 ± 0.66	6.45 ± 0.77	6.50 ± 0.67

^1^ Overall, I am satisfied with the ease of completing the tasks in this scenario. ^2^ Overall, I am satisfied with the amount of time it took to complete the tasks in this scenario. ^3^ Overall, I am satisfied with the support information (digital help, messages, and documentation) when completing the tasks.

**Table 7:** Summative usability evaluation success rates by clinical role and NICU experience.

No	Category	ALL	Clinical Role		NICU Staff Experience	
			Physician	Nurses	≥8 Years	<8 Years
1	Multi-functional neonatal incubator	95.64%	94.00%	95.73%	96.15%	94.62%
2	Neonatal incubator	94.63%	94.07%	94.32%	95.22%	93.38%
3	Patient monitoring	98.33%	93.33%	100%	100.00%	96.67%
4	Radiant warmer	96.67%	93.33%	97.78%	96.67%	96.67%
5	Phototherapy	98.33%	100%	97.78%	96.67%	100.00%

**Table 8:** Statistical analysis of task load and SUS scores according to clinical role.

Questionnaire items		All Mean ± SD	Group1 ^1^ Mean ± SD	Group2 ^2^ Mean ± SD	Mann– Whitney U	Z	Effect Size (r)	*p*-Value
NASA TLX	Mental Demand	9.75 ± 9.934	9.5 ± 10.395	10.0 ± 10.541	34.5	−0.27	0.060	0.800
	Physical Demand	9.25 ± 12.577	11.5 ± 11.797	7.0 ± 14.181	26.5	−1.013	0.227	0.349
	Temporal Demand	9.5 ± 13.775	10.5 ± 14.615	8.5 ± 14.347	25	−1.171	0.262	0.306
	Performance	10.5 ± 9.605	14.5 ± 10.124	6.5 ± 8.182	35	−0.257	0.057	0.866
	Effort	11.75 ± 12.07	13.0 ± 15.492	10.5 ± 8.960	35.5	−0.181	0.040	0.866
	Frustration	10.25 ± 22.775	7.5 ± 12.748	13.0 ± 31.198	29	−0.794	0.178	0.497
SUS		78.63 ± 15.702	80.0 ± 14.142	74.25 ± 19.114	23.5	−1.227	0.274	0.230

^1^ Group 1: Physician. ^2^ Group 2: Nurses.

**Table 9:** Statistical Analysis of Task Load and SUS Scores According to NICU Staff Experience.

Questionnaire Items		All Mean ± SD	Group1 ^1^ Mean ± SD	Group2 ^2^ Mean ± SD	Mann– Whitney U	Z	Effect Size (r)	*p*-Value
NASA TLX	Mental Demand	9.75 ± 9.934	8.0 ± 7.483	10.33 ± 10.562	49	−0.078	0.017	0.971
	Physical Demand	9.25 ± 12.577	4.0 ± 5.831	11.00 ± 13.687	33	−1.355	0.303	0.218
	Temporal Demand	9.5 ± 13.775	15.0 ± 16.432	7.67 ± 12.2259	46	−0.324	0.072	0.796
	Performance	10.5 ± 9.605	7.0 ± 7.483	11.67 ± 9.944	28.5	−1.74	0.389	0.105
	Effort	11.75 ± 12.07	11.0 ± 8.000	12.00 ± 13.14	49.5	−0.039	0.009	0.971
	Frustration	10.25 ± 22.775	2.0 ± 2.449	13.00 ± 25.677	47	−0.267	0.060	0.853
SUS		78.63 ± 15.702	86.0 ± 9.301	76.17 ± 16.605	45.5	−0.341	0.076	0.739

^1^ Group 1: NICU-experienced staff (≥8 years). ^2^ Group 2: Less-experienced NICU staff (<8 years).

## Data Availability

The data presented in this study are available on request from the corresponding author due to privacy and ethical restrictions.

## Abbreviations

The following abbreviations are used in this manuscript:

ASQ	After-Scenario Questionnaire
FDA	Food and Drug Administration
GUI	Graphical User Interface
HFE	Human Factors Engineering
IEC	International Electrotechnical Commission
IRB	Institutional Review Board
ISO	International Organization for Standardization
MFDS	Ministry of Food and Drug Safety (Korea)
NICU	Neonatal Intensive Care Unit
SUS	System Usability Scale
UCD	User-Centered Design
NASA-TLX	National Aeronautics and Space Administration Task Load Index

## Appendix A

This appendix A presents a simplified use-related risk analysis derived from the findings of the usability evaluation. In accordance with IEC 62366-1 and ISO 14971 principles, the table summarizes potential use-related risks identified during usability evaluation [36].

**Table A1:** Simplified use-related risk analysis based on usability evaluation findings.

No	Category	Potential Use Error	Potential Clinical Harm	Root Cause	Risk Control and Design Improvement
1	Warmer mode transition	Failure to activate warmer mode after opening canopy	Inadequate thermal support leading to hypothermia risk	Mental model based on conventional incubators	Improve transition feedback mode and ensure workflow-consistent interaction
2	Surgical light activation	Failure to locate or activate surgical light	Delayed procedure or reduced visibility during clinical intervention	Mismatch with typical NICU workflow and unclear navigation pathway	Improve menu structure and provide clearer navigation cues
3	Parameter adjustment	Incorrect parameter setting	Inappropriate patient management or monitoring errors	Interface complexity or unfamiliar layout	Simplify interface and improve parameter visibility
